# Seleno-amino Acid Metabolism Reshapes the Tumor Microenvironment: from Cytotoxicity to Immunotherapy

**DOI:** 10.7150/ijbs.95484

**Published:** 2024-05-05

**Authors:** Rui Liang, Aoyu Cheng, Shengxin Lu, Xiaokun Zhang, Maomao Ren, Jiayi Lin, Ye Wu, Weidong Zhang, Xin Luan

**Affiliations:** 1 Shanghai Frontiers Science Center of TCM Chemical Biology, Institute of Interdisciplinary Integrative Medicine Research and Shuguang Hospital, Shanghai University of Traditional Chinese Medicine, Shanghai, 201203, China.; 2 School of Pharmacy, Second Military Medical University, Shanghai 200433, China.; 3 State Key Laboratory for Quality Ensurance and Sustainable Use of Dao-di Herbs, Institute of Medicinal Plant Development, Chinese Academy of Medical Science and Peking Union Medical College, Beijing, 100700, China.

**Keywords:** Cancer, Selenium, Seleno-amino acid, Metabolic reprogramming, Immunotherapy

## Abstract

Selenium (Se) is an essential trace element for biological processes. Seleno-amino acids (Se-AAs), known as the organic forms of Se, and their metabolic reprogramming have been increasingly recognized to regulate antioxidant defense, enzyme activity, and tumorigenesis. Therefore, there is emerging interest in exploring the potential application of Se-AAs in antitumor therapy. In addition to playing a vital role in inhibiting tumor growth, accumulating evidence has revealed that Se-AA metabolism could reshape the tumor microenvironment (TME) and enhance immunotherapy responses. This review presents a comprehensive overview of the current progress in multifunctional Se-AAs for antitumor treatment, with a particular emphasis on elucidating the crosstalk between Se-AA metabolism and various cell types in the TME, including tumor cells, T cells, macrophages, and natural killer cells. Furthermore, novel applications integrating Se-AAs are also discussed alongside prospects to provide new insights into this emerging field.

## Introduction

Cancer continues to be a prominent global cause of mortality, but conventional treatments are frequently limited by issues such as toxicity, resistance, and adverse effects 1. In recent years, tumor immunotherapy, including immune checkpoint blockade (ICB), adoptive cell transfer, and cancer vaccination, has emerged as a promising alternative for harnessing the power of the immune system to recognize and eliminate cancer cells 2. However, despite its potential benefits, many cancer patients exhibit a low response to this treatment modality, highlighting the need for innovative strategies that can augment its efficacy 3. Many studies have shown that abnormal amino acid metabolism can affect both tumor and immune cells in the tumor microenvironment (TME), leading to tumor immune evasion 4. For example, tryptophan facilitates the survival and activity of CD8+ T cells 5. However, kynurenine, an amino acid metabolite of tryptophan, can increase the expression level of programmed cell death protein 1 (PD-1) on CD8+ T cells and mediate immunosuppression by activating regulatory T cells (Tregs) 6. Hence, inspired by these findings, strategies targeting amino acid metabolism to improve the response to tumor immunotherapy have been proposed and entered clinical trials 7.

Selenium (Se) is an essential trace element that plays a crucial role in regulating biological processes 8. Se-AAs, the organic forms of Se, are considered valuable forms for Se supplementation owing to their high safety and bioavailability compared to those of inorganic Se, such as selenite and selenate 9. Increasing evidence has indicated that Se plays an important role in cancer progression, drug resistance, and immune evasion 10. Se-AA deficiency can impair the antitumor effects of chemotherapy and radiotherapy, increasing tumor resistance 11, 12. In addition to their impact on traditional cancer treatments, Se-AAs and their metabolism have been proven to show excellent potential for tumor immunotherapy, as they significantly improve the immune response by regulating the crosstalk between tumor cells and immune cells and reshaping the TME 13.

Given the diverse functions and implications of Se-AA metabolism relevant to the TME, we summarize recent advancements in the development of Se-AAs for tumor treatment to enhance our understanding of their pharmacological mechanisms, with an emphasis on their immunomodulatory effects on different kinds of cells in the TME. Additionally, we discuss the current applications and perspectives of Se-AAs for more effective tumor treatment as a novel adjunctive therapeutic strategy, which will contribute to tumor immunotherapy developments in the future.

### Biological forms of Se

Se exists in nature in three main forms: monomeric Se, inorganic Se, and organic Se. The absorption and utilization of monomeric Se are limited. Inorganic Se exists in the valence states of +4 and +6 and is found predominantly as inorganic selenate (SeO_4_^2-^) and selenite (SeO_3_^2-^) in living organisms. However, the estimated toxic effects of inorganic Se intake were found to occur at a level of 16 µg/day, whereas the corresponding threshold for organic Se was determined to be 260 μg/day 14. Organic Se found within living organisms can be classified into two main types: Se-AAs and Se-containing proteins. Mammals and microorganisms have been observed to contain two major types of Se-AAs: selenocysteine (SeCys) and selenomethionine (SeMet) 15. The primary means by which humans acquire Se are through Se-enriched plant and animal products 16, typically in the form of SeMet from grains, yeast, and meat proteins, as well as L-Se-methyl selenocysteine (MeSeCys) found in certain plant foods such as garlic and cauliflower 12, 17. It has been reported that Se deficiency can lead to various diseases, including myocardial infarction, neurological damage, and low immunity. Therefore, understanding the metabolic transformation mechanism of Se within biological systems is essential for investigating its functional role 18, 19.

### Physicochemical properties of Se in amino acids

SeCys is formed by substituting the oxygen atom in serine (Ser) at precisely the same position as Cys in its homologous protein. The enhanced biological activity exhibited by SeCys may be attributed to the distinctive physicochemical properties of Se. A thorough comparison of the chemical structures of Cys and SeCys revealed that this disparity arises from the inherent dissimilarities between sulfur (S) and Se atoms 20.

Compared to S, molecules containing Se exhibit lower redox potentials and higher reactivity, and are susceptible to oxidation or reduction. Moreover, when comparing the thiol group to the selenol group, it is evident that the pKa value of the latter is significantly lower. This observation suggested that a larger proportion of the selenol group in SeCys undergoes deprotonation and exists in its more electrophilic state as -Se. This higher reactivity can be attributed to such a transformation. Notably, the electrostatic interaction between Se and other molecules should be emphasized 21.

Based on the above physicochemical comparison, the primary merit of SeCys, which features Se as its active center, lies in its enduring catalytic efficacy during redox reactions 20, 22, 23. Significantly, Se exhibits a distinctive and readily reversible reaction with oxygen and ROS, which is not observed in sulfur 24. Therefore, Se-AAs are more versatile than common amino acids.

### Absorption and metabolic pathways of Se-AAs

**Figure 1:**
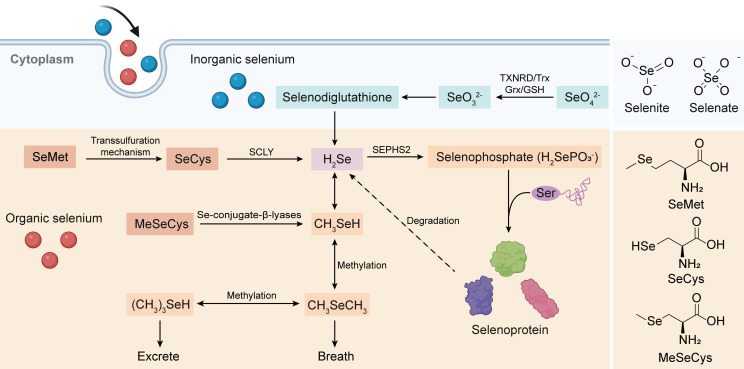
** Absorption and metabolic pathways of inorganic Se and Se-AAs**. Inorganic Se (selenate and selenite) could be stepwise reduced to produce H_2_Se, which is the bridge connecting inorganic Se metabolism and organic Se metabolism, via the intermediate selenodiglutathione. Meanwhile, Se-AAs (SeMet, SeCys, and MeCys) could also be converted to H_2_Se, followed by its transformation into selenophosphate for the synthesis of selenoproteins. Furthermore, H_2_Se is excreted through methylation reactions.

To comprehensively investigate the antitumor mechanism of Se-AAs, we conducted a thorough analysis of the complete Se metabolism pathway, which encompasses both inorganic and organic forms (Figure 1). Overall, hydrogen selenide(H_2_Se) plays a pivotal role in Se metabolism, as it serves as the nexus between two crucial metabolic pathways. Initially, Se is present in an oxidized state (selenite, Se^4+^ and selenate, Se^6+^) within inorganic substances; however, this high-valent form of Se undergoes reduction to its low-valent counterpart through the involvement of reduced glutathione and reduced nicotinamide adenine dinucleotide phosphate (NADPH) within living organisms 25. The resultant metabolite from this process, H_2_Se, actively participates in the synthesis of selenoproteins 26 while also demonstrating significant potential as an antitumor agent through the production of methylselenol (CH_3_SeH) 27. Although the various forms of Se-AAs undergo distinct metabolic pathways, they can exert antitumor effects either directly or by converting them into selenoproteins 28-30. The dietary absorption of SeCys does not directly contribute to selenoprotein synthesis; rather, SeCys is metabolized to H_2_Se by a β-cleaving enzyme (Figure 1) 26, and the substance undergoes conversion from selenide to selenophosphate 31. Simultaneously, Ser binds to a specialized tRNA, forming a serine-tRNA complex catalyzed by serine-tRNA synthetase. Subsequently, SeCys synthetase facilitates a uniquely specialized process wherein the -OH group of serine is replaced by -SeH derived from selenophosphate. This results in the formation of selenocysteine-RNA (SeCys-tRNA), which ultimately undergoes translation into selenoproteins 32.

SeMet is a popular form of dietary Se due to its exceptional bioavailability and minimal toxicity. As a Se analog of Met, SeMet can actively participate in the process of protein synthesis by substituting Met or converting it to SeCys 25. In addition, the conversion of SeMet to SeCys occurs through the transsulfuration pathway, which is also responsible for the synthesis of Cys from Met 33. The final step in the multistep process of synthesizing SeCys from SeMet involves the synthesis of selenohomocysteine and Ser followed by the elimination of SeMet via cystathionine-γ-cleaving enzymes 34. Studies have demonstrated that rats produce more SeCys when additional dietary SeMet is provided, indirectly suggesting that the storage of SeMet in proteins hinders its efficient metabolism 35.

MeSeCys, which is exclusively consumed as a dietary source and not endogenously present in the human body, is directly cleaved to CH_3_SeH by β-lyase upon ingestion. As previously mentioned, CH_3_SeH serves as a pivotal antitumor metabolite of Se; hence, MeSeCys exhibits superior biological activity *in vivo* compared to other forms of Se compounds 36. Furthermore, under the influence of β-lyase, MeSeCys can generate selenophosphoric acid, which acts as a precursor for synthesizing diverse selenoproteins and remains active when it is incorporated as a SeCys within various proteins 37.

## The crosstalk between Se-AA metabolism and various cells in the TME

The absorption and metabolism of Se have garnered significant attention within the field of oncology 38. A higher incidence of cancer, including breast cancer, lung cancer, gastric carcinoma, bladder cancer, oophoroma, pancreatic carcinoma, and melanoma, has been observed among individuals with inadequate dietary Se intake or lower plasma Se levels 12, 39, 40. Furthermore, mutations in the SeCys insertion sequence are associated with impaired lymphocyte proliferation, abnormal cytokine secretion, and telomere shortening. This finding further highlights the importance of Se in cancer treatment 41. Among the various Se compounds, the clinical application of inorganic Se compounds is limited due to their low lipid solubility, high mutagenicity, and high genotoxicity. Conversely, organic Se compounds, such as SeCys, SeMet, and MeSeCys, demonstrate enhanced cell membrane permeability and reduced side effects and systemic toxicity. Thus, organic Se holds great potential for cancer therapy 42, 43.

Se-AAs are a class of essential amino acids, and their metabolism has been implicated in numerous tumors. Together with their direct involvement in regulating various signaling pathways crucial for tumor growth and survival, several studies have highlighted the active roles of Se-AAs in remodeling the TME by modulating the crosstalk between immune cells and tumor cells. Se-AAs can activate multiple enzyme systems in lymphocytes and enhance the activity of immune cells, such as NK and T cells. Se-AAs further stimulate the secretion of lymphocyte factors with immunomodulatory effects 44, 45. Moreover, Se-AAs exhibit the potential for improving the functionality of immunoglobulin M (IgM), immunoglobulin G (IgG), and other antibodies that are crucial factors in humoral immunity 46.

### The multiple mechanisms of Se-AAs on tumor cells

The effects of Se-AAs on normal tissues and cells are primarily related to the various biological functions of selenoproteins, ranging from cellular redox regulation to the biosynthesis of hormones 30. The involvement of selenoproteins in tumor cells is multifaceted, with oxidoreductase being identified as the pivotal selenoprotein 47. These oxidoreductases include glutathione peroxidase (GPx), thioredoxin reductase (TrxR), and iodothyronine deiodinase (DIO) 43. The GPx family primarily comprises GPx1, GPx2, GPx3, and GPx4. The enzymatic activity of these selenoproteins effectively counteracts oxidative stress and inhibits cell death processes induced by inflammation 48, 49. Among them, GPx1 and GPx4 exert their protective effects against lipid peroxidation by effectively neutralizing phosphatases through the action of H_2_O_2_, thereby impeding the phosphorylation cascade 50. GPx2 acts as a negative regulator of the oncogene p53, not only inhibiting its transcriptional activity and promoting the degradation of p53 proteins but also downregulating the downstream target genes of p53. Thus, it plays a vital role in regulating the cell cycle 51. GPx3 is considered a tumor suppressor due to its role in maintaining the regulation of the thromboxane biosynthesis pathway, thereby inhibiting platelet aggregation 52. In addition, SeCys-containing TrxRs are present in both the cytoplasm (TrxR1) and mitochondria (TrxR2) and play crucial roles in reducing oxidized thioredoxin, catalyzing NADPH, regulating ascorbic acid levels, and modulating metabolism. These activities ultimately contribute to tumor growth promotion and impact patient prognosis 53.

In addition to selenoproteins, Se-AAs themselves can also exert antitumor effects. SeCys, the active center of selenoproteins, can exert its effects on tumors through the regulation of selenoproteins 54. For instance, selenophosphate synthetase 2 (SEPHS2), an enzyme regulating SeCys biosynthesis, is crucial for the survival of tumor cells to detoxify Se. The depletion of SEPHS2 in tumor cells led to the accumulation of selenide, a toxic intermediate produced during SeCys biosynthesis, resulting in the inhibition of cell proliferation, loss of colony-forming ability, and cell death 11. In addition, SeCys plays a role in blocking the tumor cell cycle 55 and promoting the activation of the p38 MAPK, JNK, and ERK signaling pathways while inhibiting AKT activity, inducing DNA damage in tumor cells 56, 57. SeCys can also induce mitochondrial dysfunction and activate ROS-mediated p53 phosphorylation to facilitate apoptosis in tumor cells 58-60. At the same time, SeCys has demonstrated targeted inhibition of TrxR1 expression 61 and radiosensitizing effects 62.

Numerous studies have demonstrated the potent inhibitory effects of SeMet on the proliferation of various tumor cells, including breast cancer, prostate cancer, and melanoma cells 63, 64. SeMet exhibited remarkable selectivity toward tumor cells in comparison to normal diploid fibroblasts or primary cells of the human prostate 65, 66. Mechanistically, SeMet selectively led to an increase in G2-M cell cycle arrest in tumor cells through the phosphorylation of P-Tyr15-p34/cell division cycle 2 kinase (cdc 2) 66, and induced apoptosis by promoting poly-ADP ribose polymerase (PARP) cleavage and the generation of ROS 67 (Table 1).

MeSeCys has been shown to inhibit the proliferation of certain tumor cell lines, such as A549 68, LNCap 69, and HOP-62 cells 70. MeSeCys can also trigger apoptosis in tumor cells by promoting lipid peroxidation and ROS generation 68, as well as inhibiting the PI3K-Akt signaling pathway 70. In particular, MeSeCys exhibited the potential to normalize angiogenesis and downregulate tumor-related proteins (such as androgen receptor and estrogen receptor α), thus overcoming tumor resistance to various therapeutic approaches, including chemotherapy 70, targeted therapy 71, and androgen deprivation therapy 69 (Table 1).

### Se-AAs reprogram macrophage immune responses

**Figure 2:**
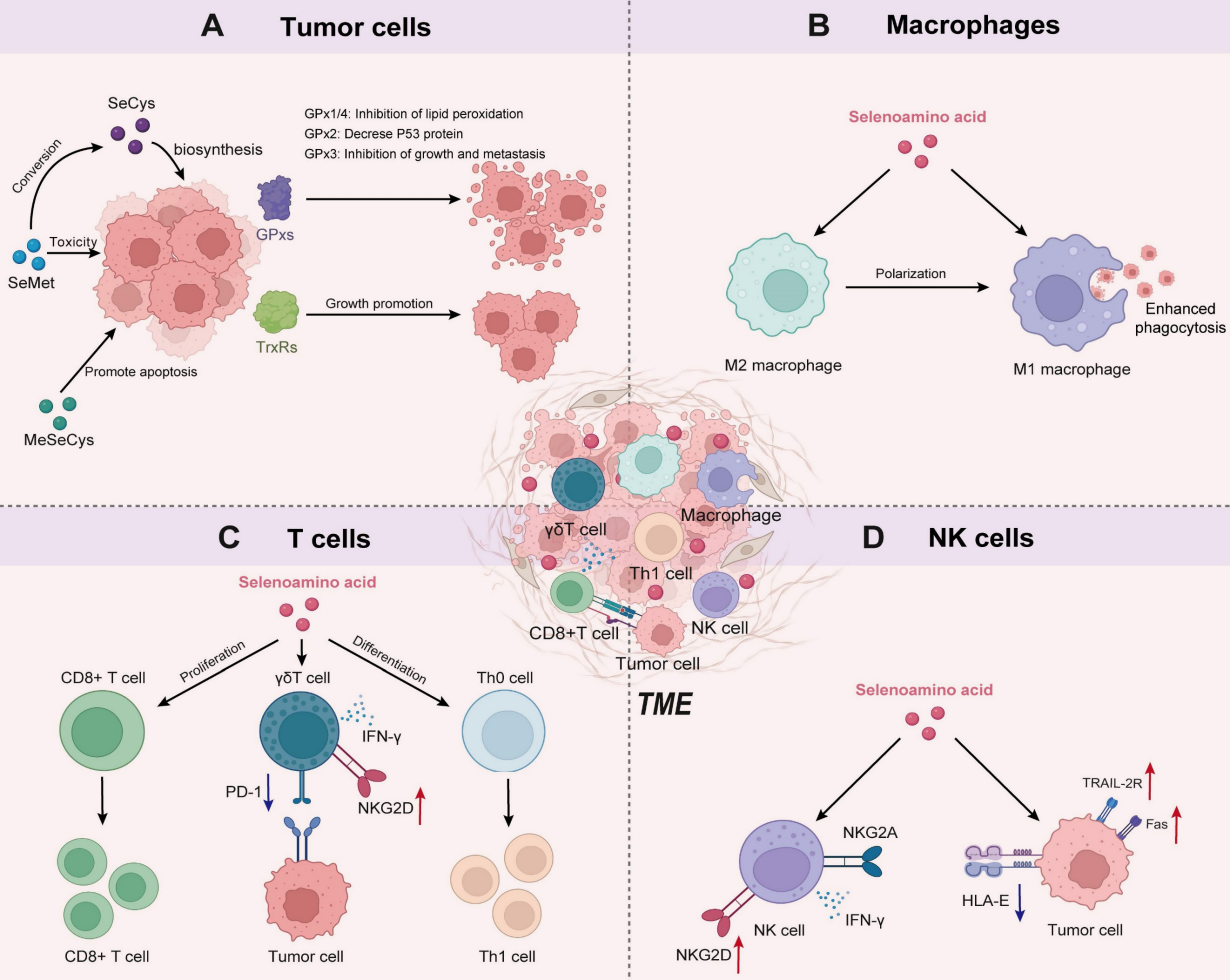
** The mechanism diagram illustrates the crosstalk between Se-AA metabolism and various cell types in the TME.** A) The multiple action of Se-AAs on tumor cell-related pathways. B) The functions of Se-AAs in programming macrophage immune responses. C) Se-AAs promote T-cell proliferation, differentiation, and cytotoxicity. D) Se-AAs boost the antitumor activities of NK cells.

Tumor-associated macrophages (TAMs) are prone to polarize to immunosuppressive M2 macrophages in the TME, thereby accelerating cancer progression and metastasis, while proinflammatory M1 macrophages exhibit preferential antitumor immune activation 72. The majority of therapeutic approaches targeting macrophages have focused on reprogramming TAMs, terminating macrophage recruitment, and interfering with TAM survival 73. However, existing approaches that depend on the blockade of the colony-stimulating factor 1/colony-stimulating factor 1 receptor (CSF1/CSF1R) axis will unavoidably compromise tissue-resident macrophages, resulting in imprecise therapeutic effectiveness 74. Chen et al. 75 designed Se nanoparticles coated with mushroom polysaccharides to restore immunity in the malignant pleural effusion of lung cancer. Their study revealed that Se nanoparticles can be gradually metabolized into selenocystine (SeCys_2_) within macrophages and educate M2 TAMs into an M1 phenotype. These results suggest that SeCys_2_ plays a key regulatory role in macrophage immune responses (Figure 2B).

### Se-AAs regulate T-cell functions

The intake of Se has been shown to impact T-cell-based adaptive immunity. Se deficiency causes atrophy of the thymus, spleen, and lymph nodes in mice, thereby inhibiting the activation and proliferation of T cells. These findings strongly indicate a close relationship between Se levels and compromised T-cell immune function 81. Se intake also promoted the proliferation and differentiation of activated CD4+ T cells into Th1 cells (Figure 2C), which are known to play a crucial role in antitumor or bacterial infection responses 81.

γδ T cells are a distinct subset of T cells that possess a unique T-cell receptor (TCR) composed of a γ and a δ chain that serves as a bridge between innate and adaptive immunity, making them crucial players in the maintenance of overall immune function 82. γδ T cells have demonstrated robust therapeutic efficacy against tumors by secreting proapoptotic molecules and inflammatory cytokines without the presence of dendritic cells. To enhance the cytotoxicity of γδ T cells, Hu et al. 83 selected Se nanoparticles to tune the antitumor ability of γδ T cells. The authors found that Se nanoparticles could increase the cancer cell-killing efficacy of γδ T cells and significantly upregulate the expression of natural killer group 2, member D (NKG2D), and interferon γ (IFN-γ) on the surface of γδ T cells while downregulating the expression of PD-1 to reduce their immunosuppressive effects (Figure 2C).

Overall, the biological effects of Se on T cells largely rely on selenoproteins synthesized from SeCys. Selenoproteins actively participate in various T-cell functions, including regulating TRC-induced calcium flux, modulating the redox activity of T cells, and linking TCR-induced activation to the metabolic reprogramming required for T-cell proliferation and differentiation 84.

### Se-AAs enhance NK-cell functions

NK cells are key effector cells in tumor immunotherapy because they can recognize specific cell surface receptors on tumor cells and pathogen-infected cells. This recognition subsequently triggers receptor-mediated cytotoxicity and cytokine production 85. NK cell cytotoxicity is attributed to the inhibitory effect of the NKG2A receptor on signaling pathways. However, within the immunosuppressive TME, NK cells encounter challenges in recognizing ligands expressed by tumor cells, which significantly impedes the therapeutic efficacy of NK cells 86. Therefore, it is important to overcome the inhibitory effects of the TME to enhance NK cell activity and tumor cell recognition. Several studies have used organic Se to effectively enhance NK cell recognition by upregulating the expression of NKG2D and NKG2DL, which is dependent on the DNA damage response pathway 87. Wei et al. 88 introduced the Sec derivative into a peptide consisting of a tumor-targeting sequence and an enzyme cleavage motif to form selenopeptide nanoparticles by self-assembly. The authors found that the combination of chemotherapy and selenopeptide-induced immunotherapy promoted reprogramming of the human leukocyte antigen E/natural killer group 2 member A (HLA-E/NKG2A) axis, activated NK cell recognition of tumors, and ultimately achieved synergistic antitumor therapy (Figure 2D) 88, 89. In addition, Se-containing complexes can also enhance therapeutic efficacy against prostate cancer by activating the death receptor (TRAIL/FasL) signaling pathway (Figure 2D) 89. These studies confirmed that Se can significantly enhance the activity of NK cells and effectively kill tumor cells via NK cells.

## Antitumor application of Se-AAs

**Figure 3:**
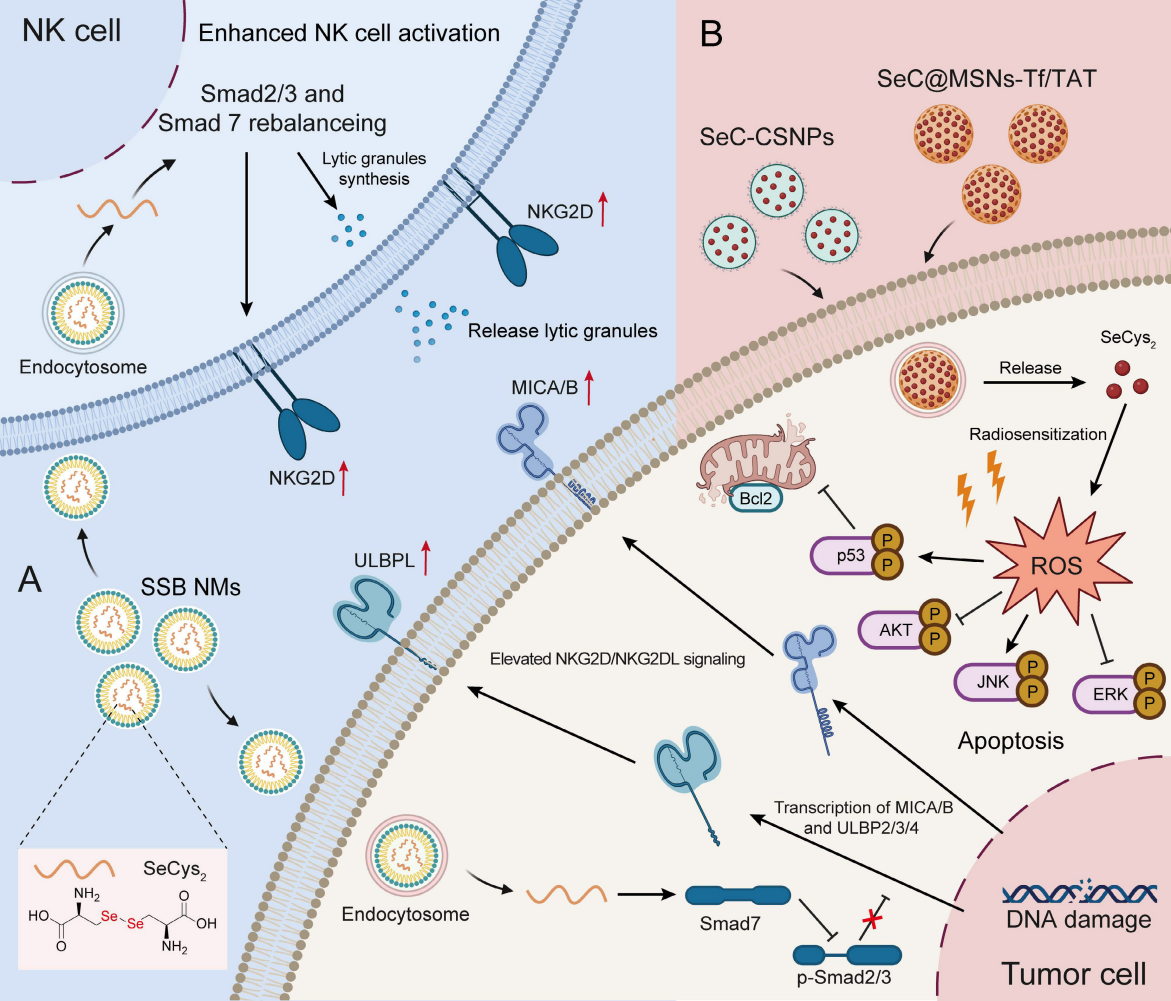
** Antitumor application of Se-AAs.** A) SSB NMs effectively suppressed the TGF-β/TGF-β RI/Smad2/3/Smad 7 signaling pathway, thereby promoting the expression of NKG2D on NK cells and NKG2DL on tumor cells, ultimately enhancing the NK cell-mediated immune response. B) The SeCys_2_ released by SeC-CSNPs and SeC@MSNs-Tf/TAT synergized with chemotherapy and radiotherapy to promote the overproduction of intracellular ROS, thereby affecting apoptosis-related signaling pathways and ultimately inducing tumor cell apoptosis.

To date, inorganic Se has several limitations due to its immeasurable toxicity, and research on Se-containing drugs has favored organic Se and Se nanoparticles. As a homolog of S, Se not only has antitumor effects but also has stronger chemical reactivity than S. Several studies have explored the utilization of diselenide bonds (Se-Se) as an alternative to disulfide bonds (S-S) in the development of chemotherapeutics, photochemotherapy and photodynamic therapy 90-92. Similar cystine, which is formed by the disulfide bond between two cysteines, SeCys_2_ readily forms a dimer due to the decreased redox potential of its selenyl group. Liu C et al. 79 constructed a nanoemulsion system (named SSB NMs) by using SeCys_2_ in combination with a TGF-β inhibitor, which resulted in a significant enhancement of NK cell-mediated antitumor efficacy against TNBC. Their findings revealed that the potentiation effect of NK cells relies on the upregulation of NKG2D signaling and NKG2D ligands (NKG2DLs). Moreover, SSB NMs effectively rebalanced the TGF-β/TGF-β RI/Smad2/3/Smad 7 signaling pathways to increase the expression of NKG2D on NK cells and NKG2DL on tumor cells (Figure 3A) 79.

Cytokine-induced killer (CIK) cell-based adoptive cell transfer has great potential in clinical cancer immunotherapy in an MHC-unrestricted manner. However, the limited *in vivo* persistence and suboptimal therapeutic efficacy of CIK cells significantly constrain their further application 93. To address these challenges, Liu et al. 80 developed an effective strategy by combining Se nanoparticles with CIK cells for synergistic immunotherapy. These authors found that SeCys_2_ was the main functional metabolite of Se nanoparticles and not only significantly prolonged the persistence of CIK cells *in vivo* but also effectively enhanced the cytotoxicity of CIK cells through upregulating the expression of NKG2D/NKG2DLs and PD-1/PD-L1 and reshaping the TME in multiple mouse tumor models (hepatic, breast and prostate tumors) 80.

In addition, numerous studies have demonstrated that SeCys_2_ can promote the overproduction of intracellular ROS. This phenomenon subsequently leads to DNA damage and influences crucial signaling pathways, such as the p53, AKT, and MAPK pathways, ultimately leading to tumor cell apoptosis (Figure 3B) 76, 77. Notably, SeCys_2_-induced ROS can also be combined with traditional chemotherapy 78 and radiotherapy to effectively overcome tumor resistance 62. For example, SeCys_2_ effectively promoted 5-FU-induced apoptosis through its modulation of Bcl2 family protein expression and the augmentation of mitochondrial membrane potential (Figure 3B). Moreover, SeCys not only exhibited radiosensitizing effects on tumors 62 but also increased radioresistance in healthy tissue by promoting cytokine secretion and augmenting the population of white blood cells while concurrently reducing bone marrow DNA inhibition 94.

## Conclusion and future perspectives

Abnormal tumor metabolism is considered a hallmark of cancer 95 and one of the main underlying factors impeding the efficacy of cancer immunotherapies 96. In addition to the well-studied glucose-dependent metabolic landscape (termed the “Warburg effect”), amino acid metabolism reprogramming in the TME is also extensively involved in the manipulation of tumor immune escape 97. Thus, targeting amino acid metabolism in tumors opens up new avenues for cancer immunotherapies by orchestrating the uptake, transport, and metabolism of amino acids, such as glutamine 98, tryptophan 99, and arginine 100. Among them, Se-AAs are crucial for maintaining the cellular oxidation-reduction balance and the immune system in mammals, and remarkable progress has been made in Se-AA metabolism reprogramming within the TME 101. With an increasing understanding of the various underlying mechanisms, targeting Se-AA metabolism has emerged as a promising therapeutic strategy with potential clinical implications.

Recently, owing to the unique physiochemical properties and pharmacological activities of Se, numerous Se-containing small molecules have exhibited decreased toxicity and improved antitumor bioactivities by incorporating Se into structural scaffolds, making them promising compounds 102. Additionally, Se-AAs have also been used as chemical handles for the synthesis and functionalization of peptides and proteins, such as metal-free/metal-catalyzed transformations, traceless chemical modifications, and protein folding 103. However, current research related to Se-AA-containing drugs, especially peptide drugs, is very scarce. Therefore, it is imperative and highly important to study the pharmacological effects of Se-AA-containing compounds in the future. Considering the potential advantages of organoselenium in medicinal chemistry, we hypothesize that the development of Se-AA-based conjugates will be a powerful strategy for generating novel tumor immunotherapy agents or adjuvants to enhance current regimens used in clinical immunology.

## Figures and Tables

**Table 1:** Antitumor mechanism of Se-AAs.

Se-AAs	Target cells	Effects	Mechanism	Ref.
SeCys	MDA-MB-231 cells	Selenoprotein production via the SLC7A11-SEPHS2 axis	Cancer cells can detoxify selenide produced in the SeCys biosynthesis pathway via the SEPHS2 protein, and overexpression of the SEPHS2 protein protects cancer cells against selenite.	11
SeCys	U251/U87/MG-63 cells	DNA damage and MAPK and AKT pathways modulation	SeCys promotes DNA damage through inducing ROS generation. SeCys causes p38MAPK, JNK and ERK activation and AKT inactivation, thereby inducing DNA damage in tumor cells. Mitochondrial dysfunction and imbalanced Bcl-2 family expression. Activation of TrxR1-targeted inhibition	55-57], [61
SeCys	A375/MCF-7/MDA-MB-231/HepG2 cells	Cell cycle arrest and apoptosis	Cell cycle arrest with reduced expression of associated proteins, including cyclins A and CDK-2. Activation of caspase-independent apoptosis. Activation of ROS-mediated mitochondrial pathway. Promotion of p53 phosphorylation	58-60
SeCys	HeLa/Caski/SiHa cells	Radiosensitization	SeCys can act as a radiosensitizer and significantly enhance ROS production in cancer cells following X-ray treatment.	62
SeCys_2_	A375/HeLa/HepG2/MCF-7 cells	Pro-apoptosis	SeCys_2_ could promote the overproduction of ROS in tumor cells, leading to DNA damage and affecting the p53, AKT, and MAPK pathways to induce apoptosis of tumor cells. SeCys_2_ enhances 5-FU-induced loss of mitochondrial membrane potential by regulating the expression of Bcl2 family proteins	76-78
SeCys_2_	NK cells and MDA-MB-231 cells	Upregulation of recognition ligands	SeCys_2_ rebalances the Smad 2/3/Smad 7 signaling pathways to increase the expression of NKG2D on NK cells and NKG2DL on tumor cells, respectively.	79
SeCys_2_	CIK cells and HepG2 cells	Enhanced persistence of CIK cells and regulation of recognition ligands	SeCys_2_ prolongs the persistence of CIK cells *in vivo* and effectively enhances the cytotoxicity of CIK cells by regulating the expressions of NKG2D/NKG2DLs and PD-1/PD-L1.	80
SeMet	LNCaP/PC-3/DU145 cells	Cell cycle arrest	SeMet results in the G2-M cell cycle by phosphorylating cdc2.	66
SeMet	A549/HepG2 cells	Pro-apoptosis	SeMet promotes glutathione depletion and induces high levels of ROS in tumor cells, further leading to apoptosis.	67
MeSeCys	A549 cells	Inhibition of tumor cell proliferation	MeSeCys induces lipid peroxidation and increases ROS generation in A549 cells.	68
MeSeCys	LNCaP cells	Inhibition of castration-resistant progression of LNCaP tumors	MeSeCys downregulates the expression of androgen receptor and prostate-specific antigen, inhibits proliferation and angiogenesis, and induces apoptosis in LNCaP tumors.	69

## Abbreviations

cdc 2: P-Tyr15-p34/cell division cycle 2 kinase
CH_3_SeH: Methylselenol
CIK: Cytokine-induced killer
CSF1/CSF1R: Colony-stimulating factor 1/colony-stimulating factor 1 receptor
DIO: iodinated thyrotropine
GPx: Glutathione peroxidase
HLA-E: human leukocyte antigen E
H_2_Se: Hydrogen selenide
ICB: Immune checkpoint blockade
IFN-γ: Interferon gamma
IgG: Immunoglobulin G
IgM: Immunoglobulin M
MSeA: Methylselenic acid
MeSeCys: L-Se-methyl selenocysteine
MTB: Mycobacterium tuberculosis
NADPH: Nicotinamide adenine dinucleotide phosphate
NKG2A: Killer Cell Lectin Like Receptor C1
NKG2D: natural killer group 2 member D
PARP: poly-ADP ribose polymerase
PD-1: Programmed cell death protein 1
ROS: Reactive oxygen species
S: Sulfur
Se: selenium
Se-AAs: Seleno-amino acids
SeCys: Selenocysteine
SeCys-tRNA: Selenocysteine-RNA
SeCys_2_: Selenocystine
SeMet: Selenomethionine
SEPHS2: Selenophosphate synthetase 2
Ser: Serine
SIRPα: Signal-regulated protein alpha
TAM: Tumor-associated macrophage
TCR: T-cell receptor
TME: Tumor microenvironment
Tregs: Regulatory T cells
TrxR: Thioredoxin reductase
